# Subjective Outcome Evaluation of a Positive Youth Development Program in Mainland China: Evidence in the Post-Pandemic Era

**DOI:** 10.3390/ijerph22040613

**Published:** 2025-04-15

**Authors:** Daniel T. L. Shek, Yi-Ting Tang, Xiaoqin Zhu, Ziqian Gong

**Affiliations:** Department of Applied Social Sciences, The Hong Kong Polytechnic University, Hong Kong 999077, China; yi-ting.tang@polyu.edu.hk (Y.-T.T.); xiaoqin.zhu@polyu.edu.hk (X.Z.);

**Keywords:** adolescents, program evaluation, client satisfaction, youth program, secondary school students

## Abstract

Background: Optimizing the development of adolescents in the post-pandemic era is an urgent concern. As an active initiative, the “Tin Ka Ping P.A.T.H.S. Project”, rooted in the “Positive Youth Development (PYD)” approach, represents a curriculum-based program aimed at fostering the psychosocial competence and well-being of adolescents. This study presents evaluation findings of the program’s effectiveness during the 2022–2023 academic year. Methods: Using a validated “36-item Subjective Outcome Evaluation scale (SOES)”, we evaluated perceptions of 2165 junior students (M_age_ = 13.43 years, SD = 0.70 years, 51.3% females) and 3287 senior students (M_age_ = 16.07 years, SD = 0.65 years, 52.8% females). Results: Replicating our previous findings before and during the pandemic, the 36-item SOES exhibited satisfactory validity and reliability in the current study. Students commonly provided favorable evaluations regarding program quality, implementer quality, and perceived benefits. Senior-grade students were more likely to perceive the program more positively. Conclusions: These replications underscore the generalizability of the robustness of the 36-item SOES as a reliable evaluation measurement tool across different time periods and research backgrounds. The participants’ positive responses indicated the positive impact of the “Tin Ka Ping P.A.T.H.S. Project” in enhancing the psychosocial competence and well-being of the students across background contexts and time periods.

## 1. Introduction

The Bellagio Study Group on Child Survival [1] appealed to the importance of optimizing child and adolescent health and development. Regrettably, as Bhutta and colleagues [2] highlighted, despite the compelling evidence and calls for change, the implementation of viable measures remains deficient, leaving us with a mere fraction of a decade to realize the aspirations encapsulated within the Sustainable Development Goals (SDGs) by 2030. Adding to the complexity, the global outbreak of the COVID-19 pandemic has exacerbated the already dire state of health and well-being among young individuals [3]. In the pandemic and post-pandemic periods, adolescents face significant challenges across multiple dimensions that can compromise their health [4,5]. It is imperative, therefore, to prioritize prevention strategies that foster positive adolescent development. Embracing this vision, the concept of “Positive Youth Development (PYD)” is gaining increasing attention and implementation [6,7].

As a pioneering study utilizing the PYD approach within the Chinese context, there is accumulating supportive evidence indicating the effectiveness of the “Tin Ka Ping P.A.T.H.S. Project” in positively nurturing the psychosocial competence and well-being of young people in a school-based setting [8]. As a valuable source of information for evaluating project effectiveness, the subjective outcome evaluation approach provides a reference and insightful understanding for project implementation and improvement. To gain a comprehensive understanding of the program’s effectiveness over time, further evaluations are warranted. In this paper, we present evaluation findings utilizing the “client satisfaction” approach based on the perspective of the participating students.

### 1.1. Adolescent Development in the Post-Pandemic Era and Beyond

The COVID-19 pandemic has profoundly affected the lives of young people in multiple ways [4]. In recent research, adolescents continued to exhibit increased levels of symptoms related to depression, anxiety, social anxiety, and family issues even after the pandemic [9,10]. Jen et al. [11] investigated the social and emotional challenges faced by 2849 adolescents residing in Asia (including mainland China and Hong Kong) and the United Kingdom, both during and in the aftermath of the pandemic. These adolescents self-reported a prominent sense of loneliness, as revealed by the survey. Other behavioral issues of significant concern include internet addiction [12] and substance abuse [13]. Focusing on Chinese adolescents, in their recent study, Ding et al. [14] observed that youths in China reported lower emotional intelligence, less social support, and more stressful life events, indicating limited adaptive coping strategies and heightened stress in this population as they enter the post-pandemic era. As such, how to cultivate and promote psychosocial competencies in the youth population entering the post-pandemic era is a burning issue.

Building upon efforts to promote the health and development of adolescents, an increasingly embraced view among educators is that interventions should not solely focus on addressing deficits or problems but should instead optimize the developmental trajectories of each adolescent [15,16]. The pandemic has underscored the importance of prioritizing the cultivation of comprehensive abilities, holistic development, and well-being in youths, such as self-regulation skills [17], resilience [18], emotional regulation [19], and self-efficacy [20].

### 1.2. Positive Youth Development (PYD) Approach

An approach to youth development known as “Positive Youth Development (PYD)” has attracted increasing attention from educators, researchers, policymakers, and decision-makers as its developmental philosophy holds promise for realizing the aforementioned vision and aligning with the initiatives proposed by the World Health Organization [21] that every young individual should have the opportunity to fully realize their potential. The term “positive youth development” or the concept itself encompasses various disciplines, including but not limited to developmental psychology, education, sociology, social work, and public health, thus covering numerous workflows [22]. Various frameworks and models have been proposed based on the inspiration of the PYD philosophy. In their recent work, Shek and colleagues [23] summarized several representative and widely utilized PYD models, including the 40 developmental assets proposed by Benson [24], the 5Cs synthesized by Lerner et al. [25], the 15 PYD constructs defined by Catalano et al. [26,27], and the social–emotional learning framework embraced by Tolan et al. [28]. After reviewing and synthesizing different works in the PYD field, Benson and colleagues [22] summarized six important principles that have achieved consensus in the field: (1) all adolescents possess the potential to grow positively; (2) a developmental trajectory in a positive manner can be achieved when adolescents are embedded in environments that promote their positive development; (3) positive youth development can be further facilitated when adolescents engage harmoniously in diverse and enriching relationships within their environments; (4) these harmonious relationships and environments benefit all adolescents; (5) the community is the key system for adolescents’ positive development in all environments and ecosystems, determining the feasibility and transferability of development; (6) adolescents are the agents and primary participants in their own development.

Indeed, a substantial body of empirical research provides robust support for the effectiveness of the PYD approach in promoting positive growth in young people and achieving multidimensional positive impacts and outcomes. In a meta-analysis that reviewed 82 school-based PYD programs involving a total of 97,406 young people [29], a follow-up period ranging from six months to 18 years post-intervention confirmed the promotive effects of PYD methods and interventions on adolescent developmental outcomes. Specifically, these adolescents exhibited significantly better performance in terms of social and emotional skills, attitudes, and well-being indicators than did those in the control group [29]. In another systematic review [30], a total of 23 school-based PYD programs were examined and found to effectively enhance various intrinsic psychological predictors of adolescent well-being, such as self-worth and decision-making abilities, as well as their social skills, including the development of social confidence. Additionally, seven of these PYD programs also identified the promotion of adolescents’ ability to make healthy choices as one of their project goals. The cultivation of these skills for healthy decision-making encompassed areas such as drug refusal skills and the adoption of healthy eating behaviors.

The above-mentioned PYD programs have demonstrated promising results, thereby bolstering scholars’ and educators’ confidence in the PYD philosophy and its approaches. Indeed, PYD initiatives are increasingly transitioning from their traditional implementation in Western nations to a growing number of non-Western contexts. As highlighted in a recently published handbook [31], PYD models are now gaining momentum beyond the United States and Western Europe. For instance, a newly developed 7Cs model of PYD—an extension of the foundational 5Cs model [25]—has been empirically validated in youth populations in Malaysia [32], India, Indonesia, and Pakistan [33]. Furthermore, preliminary empirical evidence has begun to demonstrate the effectiveness of PYD programs in promoting positive developmental outcomes among young individuals in previously under-researched regions and nations, such as Jamaica [34], Slovenia [35], and South Africa [36].

As noted previously, while the PYD framework has enjoyed relatively extensive development in Western contexts, its conceptual models and intervention approaches remain comparatively novel within Chinese contexts. Prior to PYD’s introduction, youth development paradigms in Chinese contexts, such as mainland China and Hong Kong, were predominantly characterized by deficit-remediation models, which focused primarily on behavioral correction and problem prevention [37,38], and academic-achievement orientation, which emphasized scholastic performance as the central developmental metric [39,40]. The PYD framework’s strength-based perspective has introduced effective intervention strategies that target holistic growth across multiple domains of psychological competencies. As highlighted in a recent scoping review of studies on mental health prevention and intervention strategies in school settings in China [41], the development of comprehensive interventions integrating multiple strategies from both PYD and positive psychology frameworks offers a promising direction for the field.

The current PYD movement is thriving in a global context; however, a closer examination of the literature reveals that the application of PYD in the Chinese context is still lacking. In the studies reviewed by Taylor et al. [29], over half of the studies (n = 44) were conducted within the United States, while the remaining 38 studies from countries outside the United States did not include China. Similar regional characteristics were observed in the work of Curran and Wexler [30]. Among the 23 PYD programs included in the review, only three studies reported projects implemented in Asia (Hong Kong), while the remaining studies were conducted in Western countries; notably, two of these studies were predecessors of the project reported in this study, namely the “P.A.T.H.S. Project”, which was implemented in Hong Kong. Therefore, apart from the “P.A.T.H.S. Project”, Curran and Wexler’s systematic review [30] identified only one study reporting a PYD program implemented within a Chinese cultural context. This particular program aimed at developing leadership skills among secondary school students to promote their self-efficacy and self-esteem. However, the comparative analysis between the experimental and control groups indicated that this leadership training program did not yield significant effects [42]. Consequently, there is a notable research gap regarding the application and implementation of PYD approaches within the context of China.

### 1.3. The “Tin Ka Ping P.A.T.H.S. Project” (“TKP P.A.T.H.S. Project”)

The “P.A.T.H.S. Project”, known as “Positive Adolescent Training through Holistic Social Programmes”, serves as a pioneering PYD initiative within the context of China. This project is rooted in the fundamental framework put forth by Catalano et al. [26,27], which outlined a comprehensive structure consisting of 15 constructs related to PYD. The principal aim of this project is to cultivate and advance the holistic development of adolescents by fostering a diverse array of qualities. These qualities encompass competencies, self-efficacy, self-determination, recognition of positive behavior, resilience, positive identity, bonding, prosocial involvement, acquisition of prosocial norms, spirituality, and belief in the future. Originally developed as a curriculum-based youth program targeting Hong Kong students in junior high schools, the project embraced an experiential learning pedagogy that featured reflective practices, group discussions, role-playing, and various other interactive learning strategies. Since the 2005–2006 academic year, the “P.A.T.H.S. Project” has been implemented across about 280 secondary schools over eight subsequent academic years. It was later implemented as a community-based PYD program. Diverse effective evaluation methods have provided gratifying results for the positive impact of the project on young people in Hong Kong.

The success of the project in secondary schools and communities in Hong Kong has attracted financial support from the “Tin Ka Ping Foundation”, enabling the project’s continued expansion. As a result, the project was modified and introduced to mainland China under the name “Tin Ka Ping P.A.T.H.S. Project”. Starting from the 2011–2012 academic year, the project has been piloted in four cities in the East China region. A comparative study examined the impact of the modified project in mainland China, specifically between four secondary schools participating in the project and another four not involved in the project within the same cities. The study revealed significant improvements in cognitive, social, emotional, and behavioral competencies and self-efficacy among students in the project schools after one year of project implementation, as compared to the non-project schools [43]. Importantly, no statistically significant differences in scores between those from the two types of schools before the project implementation, indicating a comparable starting point. Furthermore, subjective outcome evaluation was also conducted in the study. Notably, 96.3% of the 1056 surveyed students reported perceiving the project as helpful, indicating a positive reception [43]. The comprehensive evidence derived from both objective and subjective outcome evaluations strongly corroborates the effectiveness of the adapted project in mainland China.

These promising findings and positive outcomes have paved the way for the larger-scale implementation of the “Tin Ka Ping P.A.T.H.S. Project” in mainland China. During the 2014–2015 academic year, over 450 teachers underwent intensive training through four training workshops/programs. These trained teachers subsequently became active implementers within the project. In the 2015–2016 academic year, the project was further expanded to 30 secondary schools across 25 cities in mainland China. Based on responses from a total of 7289 students who received the curriculum adapted to the project, the vast majority of the sample responded positively [44].

Further, employing a pre–post test quasi-experimental design, a study compared a total of 539 experimental subjects who were junior high school students from project schools and 505 control subjects who were students from non-project schools in terms of PYD attributes, developmental problems, and well-being [45]. The study observed significant improvements in all perceived PYD attributes among experimental subjects compared to the control subjects. Moreover, experimental subjects showed lower problem behavior and psychological morbidity than control subjects. In another study (N = 20,480), subjective outcome evaluation reaffirmed the role of the project in promoting and enhancing positive development among students during the two-year implementation period of the 2016–2017 and 2017–2018 academic years [46]. Notably, over 90% of students acknowledged benefiting from various aspects of the project, such as resilience, self-confidence, and empathy.

### 1.4. Subjective Outcome Evaluation on Program Effectiveness

As the “TKP P.A.T.H.S. Project” is gradually being implemented on a larger scale in mainland China, it becomes necessary to use more convenient, time-saving, cost-effective, and feasible evaluation strategies and methods. The “subjective outcome evaluation (SOE)” is recognized as an evaluation measurement strategy and method that can achieve this goal. Conceptually, SOE is rooted in capturing individuals’ subjective perceptions, experiences, and viewpoints to evaluate project outcomes [47]. While traditionally associated with qualitative methods (e.g., interviews, focus groups) that provide an in-depth understanding of individual opinions and experiences [48,49], SOE’s operationalization also incorporates validated quantitative measures. These standardized instruments quantitatively capture self-reported perceptions and attitudes regarding program outcomes while preserving their inherently subjective and qualitative nature. This methodological approach, that is, employing psychometrically sound quantitative tools for self-report assessment, mirrors established practices in educational and social program evaluation, where quantified subjective measures (e.g., satisfaction surveys, perceived benefit scales) [50,51] complement traditional qualitative SOE. Such methodology reflects both the practical constraints of large-scale implementation and the need for rigorous, comparable outcome data. The subjective outcome evaluation approach has commonly been used in mental health and allied professionals.

Utilizing the “client satisfaction” approach, a “36-item Subjective Outcome Evaluation Scale (SOES)” has been developed to assess the experiences and perceptions of end-users regarding the “TKP P.A.T.H.S. Project” [46]. The “client satisfaction” approach is an SOE strategy that is commonly applied to evaluate the satisfaction and feedback of clients or service recipients regarding specific programs, services, or interventions [52]. It involves directly gathering firsthand data from clients to gain insights into their perspectives, experiences, and opinions concerning the project’s effectiveness, quality, and impact, which is commonly achieved through quantitative methods [52,53]. In education research, for example, it is commonly applied to capture students’ subjective opinions and satisfaction levels regarding aspects such as activity settings and expectations [54]. The 36-item SOES comprises three subscales designed to measure participants’ perceptions of (1) program quality (10 items), such as curriculum design, focusing on evaluating the perceived quality of the program’s curriculum design; (2) implementer quality (10 items), such as teachers’ professional attitudes and teaching skills, aiming to assess the perceived quality of the instructors; and (3) perceived program benefits (16 items), exploring the perceived benefits through the project, particularly related to the enhancement of social relationships among participants.

While the SOES has been found to be valid and reliable [45,46], there is a need to replicate its psychometric properties. Replicability serves as the cornerstone of developmental and educational psychology research [55]. Each young person is unique and possesses distinct experiences. As such, especially given the variability of students and their environments, it is essential to address issues of generalizability in developmental and educational research. One prominent advantage of a replication study is to increase the generalizability of original findings and establish an accumulative research process whereby results undergo repeated checks and theory modifications [56], thus establishing scientific credibility [57]. As such, the replication of psychometric properties of the SOES becomes particularly crucial when transitioning from pre-pandemic to post-pandemic contexts. The pandemic’s manifested and potential impacts on young people may have influenced their perception of self and the world, altering their developmental needs and expectations of educational activities. Replicating the psychometric properties of SOES allows us to evaluate its reliability as well as validity in this unique context, which is vital to ensure that the evaluation instrument remains applicable and trustworthy. Furthermore, in youth development and education programs, replication research informs stakeholders about which results have been successfully replicated under specific circumstances [55]. Replicating findings demonstrates the rationality and effectiveness of intervention across time and contexts. Conversely, unsuccessful replication reveals the limitation of intervention within specific times, environments, and populations, prompting further research questions while preventing unnecessary resource costs.

### 1.5. The Present Study

**Figure 1:**
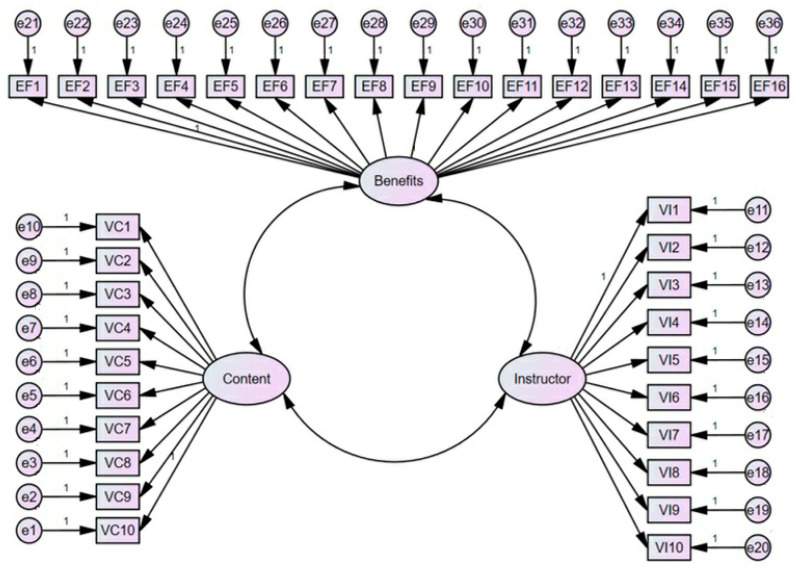
Structure of the 36-item Subjective Outcome Evaluation Scale.

This study had two objectives. First, it attempted to confirm the validity and reliability of the “36-item Subjective Outcome Evaluation Scale (SOES)”. Drawing from previous research [46], we proposed several hypotheses:

**Hypothesis** **1a:** *The 36-item SOES would exhibit factorial validity, with support for the invariance of its three-factor structure, encompassing “perceived program quality”, “perceived implementer quality”, and “perceived program benefits” (Figure 1).*

**Hypothesis** **1b:** *The 36-item SOES would exhibit convergent validity.*

**Hypothesis** **1c:** *The 36-item SOES would exhibit discriminant validity.*

We expected that the scale would show criterion-related validity with three criterion measures and proposed the specific hypotheses as follows:

**Hypothesis** **1d:** *The 36-item SOES scores would positively correlate with the tendency to recommend the project to peers.*

**Hypothesis** **1e:** *The 36-item SOES scores would positively correlate with the intention to participate in similar projects in the future*.

**Hypothesis** **1f:** *The 36-item SOES scores would positively correlate with the overall satisfaction level*.

**Hypothesis** **1g:** *The total scale and subscales would possess adequate reliability.*

The second objective is to examine the views of the participants on the program, including its content, teachers, and benefits. We also sought to investigate potential variations in students’ evaluations of the project based on their grade levels, specifically comparing junior and senior secondary participants. Building upon our prior research [46], we proposed two expectations for this study as follows:

**Hypothesis** **2:** *There would be a significant majority of participants (over 80%) who would deliver positive evaluations*.

**Hypothesis** **3:** *Junior students would evaluate more positively in comparison to their senior counterparts.*

## 2. Materials and Methods

### 2.1. Participants and Procedures

To assess students’ subjective perceptions of the “TKP P.A.T.H.S. Project” in the current study, the procedure employed in the previous work [46] was followed. A total of eight schools that were involved in the project were randomly selected for the evaluation. The teachers, who served as the implementers of the project, invited students who had participated in the project courses to voluntarily complete the SOE scale. The principles of voluntary, anonymity, and confidentiality were ensured throughout the implementation process. Every invited student agreed to complete the questionnaire, with prior parental consent for their participation in the project and project evaluation studies. In the 2022–2023 academic year of the post-pandemic era, we collected a total of 5654 valid questionnaires from students. The sample included junior (Grades 7–9) and senior (Grades 10–12) students. Specifically, we obtained 2165 responses from junior students (M_age_ = 13.43 years, SD = 0.70 years, 51.3% females), while senior students contributed 3287 responses (M_age_ = 16.07 years, SD = 0.65 years, 52.8% females).

### 2.2. Measures

The 36-item SOE scale, which was validated by Zhu and Shek [46] in their previous work, was employed. To evaluate the first two aspects of the program, “perceived quality of program” and “perceived quality of implementer”, students responded on a six-point Likert scale (1 = “*strongly disagree*”, 6 = “*strongly agree*”) to state their agreement. To evaluate the perceived program benefits, students provided their responses on a five-point Likert scale (1 = “*strongly disagree*”, 5 = “*strongly agree*”). To evaluate the concurrent validity of the SOE scale, three additional items were added to the questionnaire. The first two items assessed the tendency of participants to recommend the project to their peers and their intentions to participate in similar projects using a four-point Likert scale (1 = “*definitely will not*”, 4 = “*definitely will*”). The third item assessed the overall satisfaction level with participation experience and employed a six-point Likert scale (1 = “*very dissatisfied*”, 6 = “*very satisfied*”).

### 2.3. Analytical Strategy

To examine the psychometric properties of the SOE scale, we performed confirmatory factor analyses and invariance tests using structural equation modeling (SEM) via Mplus version 8. An examination of the sample kurtosis and skewness of responses across all items was first undertaken. If the absolute values of kurtosis and skewness exceeded the critical thresholds of 7 and 2, respectively, indicating the presence of non-normally distributed responses, then, the maximum likelihood estimator (ML) with a bootstrapping approach would be employed. This would involve 2000 resampling iterations to address the bias resulting from deviations from normality in the dataset [58].

The initial analysis focused on the supposed framework of the SOE scale, which included three factors (program quality, implementer quality, and program benefits). This analysis was performed on both the total sample (Model 1) and six sub-samples, which encompassed (1) junior students, (2) senior students, (3) male participants, (4) female participants, (5) samples with odd case numbers, and (6) samples with even case numbers (Models 2–6).

The model fit was evaluated using the following indices: “Comparative Fit Index (CFI)”, “Tucker-Lewis Index (TLI)”, “standardized root-mean-square residual (SRMR)”, and “root-mean-square error of approximation (RMSEA)”. The following evaluation criteria were adopted to indicate an acceptable fit of the data to the model: CFI and TLI should be 0.90 or higher, while SRMR and RMSEA should be 0.08 or lower [59]. Additionally, we examined changes in CFI (∆CFI) to determine whether invariance hypotheses could be accepted. Specifically, a change no larger than 0.01 indicates the consistency of measurement invariance across the six sub-samples [60].

Moreover, the study examined convergent, discriminant, and concurrent validities of the total sample. We examined the convergent validity based on the “average variance extracted (AVE)” value, with a threshold of 0.50 or higher indicating robust convergent validity [61]. Discriminant validity was established when the AVEs of the two latent factors exceeded the shared variance [62]. In addition, we conducted a series of regression analyses to examine the concurrent validity, where the item scores of three external criteria were predicted based on the factors of the SOE scale.

Subsequently, the reliability of the SOE scale and the three subscales was evaluated using the total sample. The assessment involved examining the “composite reliability (CR)” of the scale, with a desirable value of 0.70 or higher indicating good reliability [63]. Additionally, we calculated both interitem correlations and Cronbach’s alphas to examine the internal consistency of the scale [64,65].

Lastly, a one-way multivariate analysis of variance (MANOVA) was conducted on SPSS 26.0 to determine any significant differences in perceived program and implementer quality as well as program benefits between junior and senior students.

## 3. Results

### 3.1. Psychometric Properties of the 36-Item SOES

Some items failed to meet the assumption of normal distribution with absolute values of kurtosis between 0.36 and 10.28 and absolute values of skewness between 0.91 and 2.57; therefore, the maximum likelihood estimation with 2000 resampling iterations was used [62]. The three-factor model demonstrated an acceptable fit (CFI ≥ 0.94; TLI ≥ 0.93; SRMR ≤ 0.03, and RMSEA ≤ 0.06; Table 1). This consistency in fit was observed across the total sample and all sub-samples, thereby validating the proposed three-factor structure of the SOE scale.

The invariance test results for the three-factor structure are presented in Table 2. Model 8, which was not constrained by equality, demonstrated a good model fit (χ2_(1182)_ = 14,992.54, CFI = 0.94, TLI = 0.94, SRMR = 0.03, RMSEA = 0.06), indicating configuration invariance across grades. The negligible change in CFI (∆CFI = −0.001) between Model 9, with equal factor loadings, and Model 8 supported weak factorial invariance. Further, the application of equal constraints to the item intercepts in Model 10, resulting in a CFI change (∆CFI = −0.001) compared to Model 9, can also be disregarded, endorsing strong invariance across grades. Finally, in Model 11, equality constraints were imposed on the measurement residuals. However, the support for strict invariance across grades was not significant, with a CFI change of −0.033. Nevertheless, the strict factorial invariance across genders and case number sub-samples was supported, with relatively small changes in CFI in Model 15 (∆CFI = −0.002) and Model 19 (∆CFI = −0.002), respectively. Results regarding the configural, weak factorial, and strong factorial invariance tests between genders (Model 12–Model 14) and case number sub-samples (Model 16–Model 18) also supported the measurement invariance. These findings generally support Hypothesis 1a.

The convergence, discriminant validity, and reliability of the SOE scale based on the total sample are presented in Table 3. The AVE estimates for each sub-scale stood at 0.70 (program quality), 0.81 (implementer quality), and 0.76 (program benefits), signifying that a substantial portion of variance is attributable to the latent factors within the measured items. There were significant moderate-to-strong correlations between perceived quality of program and implementer (*r* = 0.86, *p* < 0.001), program quality and perceived benefits (*r* = 0.65, *p* < 0.001), and implementer quality and perceived benefits (*r* = 0.56, *p* < 0.001), reinforcing the SOE scale’s robust convergent validity (Hypothesis 1b).

We examined the discriminant validity by comparing the AVEs of each pair of factors to the squared correlation coefficient (*r*^2^) between the factors. Based on the correlation coefficients (*r*) between each pair of factors calculated in the study, i.e., 0.86, 0.65, and 0.56, the corresponding shared variances (i.e., *r*^2^) were 0.74, 0.42, and 0.31, respectively. For the “program quality—implementer quality” pair, the AVE of program quality (0.70) was found to be smaller than their shared variance (0.74). This suggested that there may be some overlap or lack of complete distinctiveness between the two factors. For the remaining pairs of factors, the AVEs were greater than their respective shared variances. To sum up, with the exception of program quality and implementer quality, the findings imply acceptable discriminant validity of the scale, thus partially supporting Hypothesis 1c.

Regression analyses were conducted to examine the predicting role of SOE factors to the three external criteria, which serve as indicators of the SOE scale’s concurrent validity. The findings were presented in Table 4 and indicated that the program quality (*β* = 0.48, *p* < 0.001) and program benefits (*β* = 0.16, *p* < 0.001) significantly predicted participants’ inclination to endorse this project to their peers, whereas implementer quality did not emerge as a significant predictor. Regarding the intent to engage in similar future initiatives, the SOE factors also demonstrated predictive value with project quality (*β* = 0.43, *p* < 0.001) and program benefits (*β* = 0.25, *p* < 0.001), while implementer quality did not exhibit significant influence. All three SOE factors (i.e., “program quality”, “implementer quality”, and “program benefits”) significantly influenced participants’ overall satisfaction (*β* = 0.47, 0.12, and 0.13, *p*s ≤ 0.001). These insights partially supported Hypotheses 1d–1f, highlighting that in the context of the post-pandemic era, perceived program quality and program benefits were more likely pivotal in predicting the likelihood of participant recommendations and engagement, as well as overall satisfaction.

The calculations of CR based on the factor loadings obtained from the CFA conducted on the full sample yielded CR values of 0.96, 0.98, and 0.98 for the three factors, respectively (Table 3). These figures affirmed the scale’s robust reliability. Furthermore, the high Cronbach’s alphas for all subscales, exceeding 0.97, along with average inter-item correlations between 0.75 and 0.82 (Table 3), gave support to the scale’s strong internal consistency. Thus, Hypothesis 1g was substantiated.

### 3.2. Subjective Evaluation of Program Effectiveness

Table 5, Table 6 and Table 7 show the ratings of participants in the two grade-level sub-samples as well as the total sample on the three subscales of the SOE scale and the satisfaction ranking. Specifically, across the three study samples, students provided mean scores exceeding 5 on all items of the two subscales, “Perceived Program Quality” and “Perceived Implementer Quality”, which were assessed using 6-point Likert-type scales (range: 1–6). Similarly, for the subscale “Perceived Program Benefits”, assessed using a 5-point scale (range: 1–5), the mean scores on nearly all items also exceeded 4.

Furthermore, over 93% of the students expressed positive responses towards program quality. Additionally, over 96% rated the clarity and appeal of the curriculum objectives positively. The vast majority (over 97%) expressed positive views about the program implementers. In terms of perceived benefits, most responses were positive, with over 91% indicating that students benefited in various aspects from participation, such as enhanced cognitive skills, self-awareness, and holistic development. These favorable evaluations lend support to Hypothesis 2.

### 3.3. Differences in Perceptions Across Grade Levels

Interestingly, contrary to our expectation, although significant differences were observed in perceptions between junior and senior students (Wilk’s λ = 6.05, *p* < 0.001, *η_p_*^2^ = 0.007; Table 8), the younger cohort (junior high) reported a lower level of positive perception of program quality (M = 5.18, SD = 0.84) compared to their senior counterparts (M = 5.37, SD = 0.82, *F* = 8.41, *p* = 0.004, *η_p_*^2^ = 0.002). Similarly, the level of positive feedback regarding the quality of implementers was lower for junior students (M = 5.37, SD = 0.78) than for seniors (M = 5.53, SD = 0.75, *F* = 14.07, *p* < 0.001, *η_p_*^2^ = 0.003). However, no significant difference was observed in perceived benefits between the two grade-level sub-samples. Hypothesis 3 was not supported by these findings. Nevertheless, it is important to highlight that the evaluations from both junior and senior high school students were generally favorable, with the differences between the groups being relatively small.

## 4. Discussion

Entering the post-pandemic era, effective and feasible strategies and initiatives for the comprehensive and holistic development of minors are urgently needed [2]. Hence, the PYD program, such as the “TKP P.A.T.H.S. Project”, is a timely and promising response.

Regarding the psychometric properties of SOES, consistent with previous work conducted before [46] and during [66] the COVID-19 pandemic, the developed 36-item SOES was validated to have robust psychometric properties in this study. The study revealed that the three-factor conceptual framework of the 36-item SOES, namely, “program quality”, “implementer quality”, and “program benefits”, aligned well with the data, exhibiting factorial invariance across diverse sub-samples. Moreover, the scale exhibited robust convergent and discriminant validities, along with high internal consistency among the three subscales. The two factors, “program quality” and “perceived program benefits”, were significantly positively associated with the additional satisfaction measures, which bolstered the concurrent validity of the scale. These results, consistent with previous research conducted prior to the pandemic [46], highlight the validity and reliability of the 36-item SOES across different contexts, thus indicating its applicability in assessing the project’s effectiveness from client satisfaction perspectives. However, as strict invariance across grades was not significant, there is a need to replicate the findings in future.

Replicating previous observations before and during the COVID-19 pandemic [44,46,66], the results of this study once again highlighted the commonly perceived benefits and the high level of satisfaction among Chinese secondary school students participating in the “TKP P.A.T.H.S. Project”. Specifically, although students’ ratings of all items in the “Perceived Program Quality” subscale ranged from 1 to 6 points, the mean scores across all items exceeded 5 points, indicating that the vast majority of students provided highly positive ratings (over 5 points) on program quality. Indeed, in the present study, 94.63% of the surveyed junior students and 96.91% of the surveyed senior students indicated overall enjoyment of the program. The junior students who previously participated in the “TKP P.A.T.H.S. Project” vividly described in their diaries that “*it is not only a way to relax in intense study, but also an expansion and innovation in addition to life and textbook learning.*” “*I have perfected my life goals and continued to explore and develop*” [67]. They found the curriculum highly useful and educational, with inspiring learning goals, clear structure, and well-designed content. In the current study, the positive evaluations regarding the quality of implementers are particularly noteworthy. Students’ ratings on the “Perceived Implementer Quality” subscale revealed consistently highly positive evaluations, with mean scores exceeding 5 points across all items. While student ratings spanned the full range of the scale (1–6 points), this pattern demonstrates that the majority of participants rated implementer quality favorably. Indeed, we observed in this study that both junior and senior students gave affirmative responses exceeding 95% for all 10 attributes of implementer quality.

Both the perceived quality of the program and the implementer are crucial for ensuring the effectiveness of the project. Indeed, the perceptions regarding program content and implementer quality were demonstrated to be significant predictors of the perceived effectiveness of the program [44]. Therefore, it is not a surprise to note that positive evaluations exceeding 90% were received from both junior and senior secondary students for almost all 16 items related to perceived program benefits in the present study. This observation is further substantiated by the consistently high mean scores exceeding 4 points (on a 5-point scale) across nearly all evaluation items in the “Perceived Program Benefits” subscale. Among all surveyed students, the two items that received the highest positive feedback included “It has strengthened my ability to distinguish between the good and the bad”. (95.02% and 95.17%, respectively, in the two sub-samples) and “It has enriched my overall development”. (95.13% and 96.06%, respectively, in the two subgroups). The improvement in cognitive and moral competencies, as evidenced by the diaries of students who previously participated in the project, was also vividly demonstrated: “*I learned to have compassion and love for others, and I also learned to distinguish between right and wrong*”. “*I learned to help me develop my talents, and let me know what to do and what not to do. Sharpen your will, refuse bad temptations, and constantly enhance your self-control*” [67].

Additionally, the majority of surveyed students in this study expressed satisfaction with the “TKP P.A.T.H.S. Project”, with mean scores exceeding 5 points (on a 6-point scale) across all three analytical samples (the total sample and two grade-level subsamples). Specifically, 97.67% of junior secondary students and 98.49% of senior secondary students indicated their satisfaction. These results allow us to confidently conclude that participants were highly satisfied with the program. Consistent with our previous work [46,66], subjective evaluations of the project in the three aspects also emerged as significant predictors of overall satisfaction. Furthermore, a considerable number of students expressed their intention to recommend the project to their peers. Similarly, most students showed a favorable inclination toward future engagement. The program quality and the perceived benefits significantly predicted participants’ responses in these two areas. However, contrary to our pre-pandemic findings [46], the quality of implementers did not exhibit a significant predictive role in the current study, which is a result consistent with our other pandemic-period research [66].

We replicated the previous findings obtained before and during the COVID-19 pandemic in the post-pandemic period. These replications convincingly demonstrate the generalizability and applicability of the assessment instrument. Additionally, the findings of subjective outcome evaluations also underscore the impact of the “TKP P.A.T.H.S. Project” on enhancing the psychosocial competence and well-being of the participants. The experience of the pandemic did not diminish the perceived benefits, positive experiences, and favorable evaluations among students toward the program. This suggests that the program continues to be valuable and effective in the post-pandemic era, fostering and enhancing students’ abilities in various dimensions. A particularly notable finding is that nearly 95% of students acknowledged that the project significantly enhanced their ability to resist harmful influences, make informed decisions, and increase self-awareness. Moreover, the project played a guiding and assisting role in shaping their positive attitude towards the future. These essential abilities and qualities directly address the developmental needs arising from the unique context of the pandemic experience. This further strengthens the notion of fostering comprehensive abilities and promoting harmonious development in alignment with the specific needs of young individuals.

Consistent with our findings revealed during the COVID-19 pandemic [66], while contrary to previous observations before this challenging and unique time [46], we found that higher-grade students were more likely to provide positive evaluations compared to younger ones in the current study. Compared to younger students, the school closures and learning disruptions caused by the pandemic may have a greater impact on senior high school students. It can be reasoned that in contrast to junior high school students, senior high school students may have a greater need for educational continuity, social connections, profound self-awareness, and life guidance [68,69]. This may explain the observed grade differences in program quality perception and implementer quality perception in this study. Additionally, the higher stress in higher grade students may make the program more appealing to them. The instructional content design in the program and the guidance provided by well-trained implementers effectively addressed their urgent needs during the post-pandemic era. Consequently, they perceived the program more positively.

### 4.1. Theoretical and Practical Implications for PYD Research and PYD Programs

#### 4.1.1. Theoretical Implications

This study provides some theoretical implications. Firstly, positive responses among Chinese young people in the “TKP P.A.T.H.S. Project” support the applicability of the PYD approach in a non-Western context. This finding is important for understanding and promoting the cross-cultural applicability of the PYD theory. The PYD theoretical frameworks and models have mainly been developed in Western countries [23]. This study highlights the comparable universality of the PYD approach in other cultural backgrounds, which contributes to advancing cross-cultural research on PYD approaches and models and promoting the cross-regional implementation of the PYD theory. A recent scientometric analysis of PYD research published from 1995 to 2020 emphasizes the literature gap in cross-cultural conceptual integration and operational definition of PYD, although some collaborations between China and the United States were identified [70]. The “TKP P.A.T.H.S. Project” serves as an example that integrates 15 PYD constructs that originated from Western culture and incorporates the cultural values of the Chinese nation. Such an attempt enhances the compatibility of the PYD theory with the local culture. This initiative provides insights into PYD research in other cultural contexts. Our ongoing efforts to implement the PYD program in the Chinese cultural context contribute to the conceptualization of PYD and the promotion of mechanisms for PYD.

Furthermore, the replication of results demonstrated the three-factor structure of subjective outcome evaluation provides support for the conceptual framework of the “client satisfaction” approach [46]. Through the development of the SOES comprising 36 items, our study provides a client-centered evaluation framework for measuring and assessing the quality of educational programs, implementer quality, and perceived benefits from the perspective and experiences of the clients. By examining the validity of this scale, our research establishes an objective assessment system that offers a reliable tool for researchers and practitioners. Through the successful replication of previous research findings, this study further confirms the replicability of this measurement tool. This scale consistently produces consistent results across different samples and research settings, enhancing confidence in the research findings.

#### 4.1.2. Practical Implications

This study also provides several important practical implications. Firstly, the findings from the current study further support the continued implementation of PYD programs in Chinese communities. By replicating our pre-pandemic [46] and pandemic-era [66] results, this research offers further robust and reliable evidence for the effectiveness of the “TKP P.A.T.H.S. Project” across different research contexts and temporal periods. Based on the experiences of implementing the “P.A.T.H.S. Project”, Shek and Dou [8] summarized several important lessons learned, one of which highlights the need for more PYD programs to be implemented in China and promote their long-term sustainability. The implementation and demonstrated effectiveness of the “TKP P.A.T.H.S. Project” serve as important initiatives that call for the practice of PYD programs in different regions and communities in China. Corroborating this perspective, a recent scoping review [41] of 77 studies conducted in China between January 2000 and May 2024 specifically cites the “P.A.T.H.S. Project” as a prime exemplary model of an integrated PYD approach. The review’s authors emphasize that such multidimensional interventions effectively address the limitations of seeking a singular “optimal” intervention strategy while more comprehensively meeting the developmental needs of youth populations [41]. Simultaneously, we advocate for the development and utilization of educational materials that integrate the cultural background of the Chinese nation based on PYD principles. Such educational materials will help blend PYD principles with Chinese cultural values, making PYD programs more relevant to the needs and realities of Chinese youth. By providing education materials that align with Chinese cultural characteristics, this practice can enhance the acceptability and sustainability of PYD programs in China.

Secondly, this study also highlights the important role of teachers equipped with PYD-related knowledge and skills in the effectiveness of the program. In fact, Shek and Dou [8] also emphasized in their previous summary the significance of training potential teachers (implementers) of PYD programs in the implementation and positive outcomes. The endorsement of implementer quality by the participating students in the study directly acknowledges the outcomes of teacher training in this project. In teacher-centered educational and classroom models, such as in mainland China, there is a need for teachers to become the gatekeepers who drive or even change the ways of teacher–student interactions. PYD methods may not be familiar to teachers within the Chinese education system; therefore, it is important to enhance teachers’ awareness and understanding of PYD while emphasizing the importance of PYD training within the teacher community. Such training can help teachers become key figures driving or even changing the ways of teacher–student interactions. By raising teachers’ awareness and capabilities, the long-term sustainability of PYD programs in China can be ensured, further promoting the effective dissemination of the PYD programs. In a recent paper, Shek and Dou [8] further highlights the importance of teacher training in PYD programs.

Lastly, this study supports the use of the developed 36-item SOES in the effectiveness assessment of PYD programs. Methodologically, the present study replicated the previous findings, suggesting that the SOES can be confidently considered a robust and reliable assessment tool, which is capable of capturing the experiences, perceptions, and viewpoints of direct participants in project engagement. It can provide reliable and firsthand evidence of the effectiveness of the “TKP P.A.T.H.S. Project” and other PYD initiatives, addressing the pressing need for evidence-based practices in the PYD field. This contributes to identifying program strengths and areas for improvement, providing a basis for project enhancement and decision-making. As emphasized by Shek and Dou [8], program evaluation is an ongoing process, and the SOES, as an objective assessment tool that yields empirical data and information, offers the advantage of being cost-effective. These advantages render it operationally feasible for the continuous evaluation of programs. Current PYD initiatives are rapidly evolving into an emerging paradigm within global contexts, particularly necessitating novel, valid, and efficient assessment methodologies for cross-regional program implementation. Our replicated findings provide PYD practitioners across sectors with a precise, reliable, and innovative measurement tool. Furthermore, the “client satisfaction” approach has also been validated as a reliable and cost-effective evaluation strategy that can be easily applied in the fields of education and social work.

### 4.2. Limitations and Suggestions for Future Research

Several limitations have been identified in the study. Firstly, students’ subjective evaluation of the program, solely assessed upon the completion of the project, does not capture the dynamic changes in their experiences, adaptability, compliance, and benefits throughout their involvement. To address this limitation, future research could assess students’ perceptions and viewpoints at multiple time points across different project implementations. Secondly, relying solely on students’ responses in the SOES may not fully capture the richness and depth of their perspectives. Therefore, future work could adopt a mixed-method design, incorporating interviews at various stages of project implementation and utilizing additional evaluation methods, such as classroom observations, to provide a more comprehensive capture and description of the students’ experiences. Some initiatives, such as using student diaries, would be beneficial [67,71]. Thirdly, the hypotheses proposed in this study were not fully supported. We observed a relatively higher level of positive perception regarding project quality and implementer quality among older students, contrary to the anticipated findings among younger students. It is necessary for future research to compare the differences in program perception and experiences among students of different age groups to determine whether the findings from this study are context-specific, particularly related to the COVID-19 pandemic. This will help us ascertain the applicability of the study findings in other contexts.

## 5. Conclusions

In conclusion, this study successfully replicated our previous research conducted prior to and during the pandemic by evaluating the “TKP P.A.T.H.S. Project” in the post-pandemic context. The 36-item SOES as the measurement tool, developed based on the “client satisfaction” approach, has been further validated for its good validity and reliability. In the study, the majority of students expressed positive attitudes and provided favorable evaluations regarding their perceptions toward the three factors, namely “program quality”, “implementer quality”, and “perceived program benefits”, indicating the effectiveness of the project. These replications highlight the generalizability of the program’s intervention design, implementation, and evaluation measurement strategies across different time periods and contexts.

## Figures and Tables

**Table 1:** Summary of goodness of fit for CFA models.

Model	Description	χ^2^	*df*	CFI	TLI	SRMR	RMSEA (90% CI)
M1	Whole sample (N = 5654)	13,786.83	591	0.95	0.94	0.03	0.063 (0.062, −0.064)
M2	Junior (N = 2245)	5484.09	591	0.94	0.94	0.03	0.061 (0.059, −0.062)
M3	Senior (N = 3409)	10,661.23	591	0.94	0.94	0.03	0.071 (0.070, −0.072)
M4	Male (N = 2606)	6146.70	591	0.95	0.95	0.03	0.060 (0.059, −0.061)
M5	Female (N = 2846)	8879.70	591	0.94	0.93	0.03	0.070 (0.069, −0.071)
M6	Odd (N = 2827)	7748.82	591	0.94	0.94	0.03	0.065 (0.064, −0.067)
M7	Even (N = 2827)	7530.76	591	0.95	0.94	0.03	0.064 (0.063, −0.066)

CFA = confirmatory factor analysis; *df* = degree of freedom; CFI = comparative fit index; TLI = Tucker Lewis index; SRMR = standardized root mean square residual; RMSEA = root mean square error of approximation; CI = confidence interval.

**Table 2:** Summary of goodness of fit for the invariance tests.

Model	Description	χ^2^	*df*	CFI	TLI	SRMR	RMSEA [90% CI]	Model Comparison	∆χ^2^	∆*df*	∆CFI
Grade-level invariance											
M8	Configural invariance (baseline model)	14,992.54	1182	0.94	0.94	0.03	0.060 (0.059, −0.061)				
M9	Weak factorial invariance	15,163.43	1215	0.94	0.94	0.03	0.059 (0.058, −0.060)	M8 vs. M9	170.88 ***	33	−0.001
M10	Strong factorial invariance	15,393.46	1248	0.94	0.94	0.03	0.059 (0.058, −0.060)	M9 vs. M10	230.04 ***	33	−0.001
M11	Strict factorial invariance	25,223.52	1287	0.91	0.91	0.07	0.081 (0.080, −0.082)	M10 vs. M11	9830.05	39	−0.033
Gender-level invariance											
M12	Configural invariance (baseline model)	15,098.51	1182	0.94	0.94	0.03	0.066 (0.065, −0.067)				
M13	Weak factorial invariance	15,183.93	1215	0.94	0.94	0.03	0.065 (0.064, −0.066)	M12 vs. M13	85.43 ***	33	0.000
M14	Strong factorial invariance	15,303.77	1248	0.94	0.94	0.03	0.064 (0.063, −0.065)	M13 vs. M14	119.84 ***	33	0.000
M15	Strict factorial invariance	15,855.73	1287	0.94	0.94	0.05	0.064 (0.064, −0.065)	M14 vs. M15	551.95 ***	39	−0.002
Subgroup invariance (odd and even case numbers)											
M16	Configural invariance (baseline model)	14,848.61	1182	0.95	0.94	0.03	0.059 (0.058, −0.060)				
M17	Weak factorial invariance	14,894.73	1215	0.95	0.94	0.03	0.058 (0.057, −0.059)	M16 vs. M17	46.11	33	0.000
M18	Strong factorial invariance	14,930.97	1248	0.95	0.94	0.03	0.057 (0.057, −0.058)	M17 vs. M18	36.24	33	0.000
M19	Strict factorial invariance	15,639.73	1287	0.94	0.95	0.03	0.063 (0.062, −0.064)	M18 vs. M19	708.76	39	−0.002

*df* = degree of freedom; CFI = comparative fit index; TLI = Tucker Lewis index; SRMR = standardized root mean square residual; RMSEA = root mean square error of approximation; CI = confidence interval. *** *p* < 0.001.

**Table 3:** Validity and reliability of the Subjective Outcome Evaluation Scale.

Subscales	Item	FL	95% CI of FL		Mean FL	AVE	CR	Cronbach’s α	Mean Interitem Correlation
			Lower	Upper					
Perceived Program Quality					0.83	0.70	0.96	0.97	0.75
	Item 1	0.79	0.74	0.82					
	Item 2	0.88	0.86	0.89					
	Item 3	0.86	0.85	0.88					
	Item 4	0.83	0.81	0.84					
	Item 5	0.78	0.76	0.80					
	Item 6	0.77	0.75	0.79					
	Item 7	0.82	0.80	0.83					
	Item 8	0.87	0.86	0.88					
	Item 9	0.89	0.88	0.90					
	Item 10	0.87	0.86	0.88					
Perceived Implementer Quality					0.90	0.81	0.98	0.98	0.82
	Item 1	0.90	0.89	0.91					
	Item 2	0.92	0.91	0.93					
	Item 3	0.92	0.91	0.93					
	Item 4	0.92	0.91	0.93					
	Item 5	0.91	0.90	0.92					
	Item 6	0.90	0.89	0.91					
	Item 7	0.89	0.88	0.91					
	Item 8	0.87	0.85	0.88					
	Item 9	0.87	0.86	0.89					
	Item 10	0.88	0.87	0.90					
Perceived Program Benefits					0.87	0.76	0.98	0.98	0.77
	Item 1	0.87	0.86	0.88					
	Item 2	0.89	0.88	0.89					
	Item 3	0.88	0.87	0.89					
	Item 4	0.88	0.87	0.89					
	Item 5	0.88	0.87	0.88					
	Item 6	0.87	0.86	0.88					
	Item 7	0.88	0.87	0.89					
	Item 8	0.88	0.87	0.89					
	Item 9	0.84	0.83	0.86					
	Item 10	0.87	0.86	0.88					
	Item 11	0.87	0.86	0.88					
	Item 12	0.87	0.87	0.88					
	Item 13	0.86	0.85	0.87					
	Item 14	0.85	0.84	0.86					
	Item 15	0.88	0.87	0.89					
	Item 16	0.85	0.84	0.86					

FL = factor loading; CI = confidence interval; AVE = average variance extracted; CR = composite reliability.

**Table 4:** Predicting effects of subjective outcome evaluations on the three external criteria ratings.

Predictors	*B*	*SE*	*β*	95% CI of *β*		*R* ^2^
				Lower	Upper	
DV: Willingness to suggest other students to participate in the program						0.35
Program quality	0.32	0.02	0.48 ***	0.44	0.51	
Implementer quality	−0.01	0.04	−0.01	−0.09	0.07	
Perceived benefits	0.13	0.04	0.16 ***	0.08	0.24	
DV: Willingness to participate in similar programs						0.34
Program quality	0.29	0.02	0.43 ***	0.39	0.46	
Implementer quality	−0.04	0.04	−0.05	−0.12	0.02	
Perceived benefits	0.21	0.04	0.25 ***	0.18	0.32	
DV: Overall satisfaction						0.42
Program quality	0.48	0.02	0.47 ***	0.42	0.51	
Implementer quality	0.15	0.03	0.13 ***	0.06	0.19	
Perceived benefits	0.16	0.04	0.13 ***	0.05	0.20	

DV = Dependent variable, CI = Confidence interval. *** *p* < 0.001.

**Table 5:** Participants’ evaluations of program quality and implementer quality.

Items	Junior Secondary (N = 2245)				Senior Secondary (N = 3409)				Overall (N = 5654)			
	Full Responses (Options 1–6)		Positive Responses (Options 4–6)		Full Responses (Options 1–6)		Positive Responses (Options 4–6)		Full Responses (Options 1–6)		Positive Responses (Options 4–6)	
	Mean (SD)	Range	n	%	Mean (SD)	Range	n	%	Mean (SD)	Range	n	%
**Perceived program quality**												
1. The objectives of the curriculum are very clear.	5.26 (0.92)	1–6	2232	96.46%	5.40 (0.89)	1–6	3279	96.53%	5.34 (0.90)	1–6	5629	96.50%
2. The design of the curriculum is very good.	5.24 (0.93)	1–6	2236	96.42%	5.41 (0.87)	1–6	3290	96.91%	5.34 (0.90)	1–6	5631	96.71%
3. The activities were carefully planned.	5.23 (0.96)	1–6	2225	95.69%	5.40 (0.88)	1–6	3273	96.61%	5.33 (0.92)	1–6	5613	96.24%
4. The classroom atmosphere was very pleasant.	5.20 (1.05)	1–6	2230	93.81%	5.44 (0.90)	1–6	3260	96.36%	5.34 (0.97)	1–6	5613	95.35%
5. There was much peer interaction amongst the students.	5.22 (1.03)	1–6	2206	94.11%	5.38 (0.95)	1–6	3234	95.85%	5.32 (0.98)	1–6	5580	95.16%
6. I participated actively during lessons (including discussions, sharing, games, etc.).	5.06 (1.15)	1–6	2231	91.39%	5.27 (1.03)	1–6	3207	94.66%	5.19 (1.08)	1–6	5619	93.36%
7. I was encouraged to do my best.	5.06 (1.10)	1–6	2230	92.65%	5.24 (1.03)	1–6	3190	94.24%	5.17 (1.06)	1–6	5615	93.61%
8. The learning experience I encountered enhanced my interest towards the lessons.	5.15 (1.05)	1–6	2229	93.99%	5.33 (0.97)	1–6	3239	95.77%	5.26 (1.01)	1–6	5611	95.06%
9. Overall speaking, I have a very positive evaluation of the program.	5.18 (1.01)	1–6	2233	94.45%	5.38 (0.93)	1–6	3262	96.22%	5.30 (0.97)	1–6	5623	95.52%
10. On the whole, I like this curriculum very much.	5.27 (1.01)	1–6	2234	94.63%	5.46 (0.89)	1–6	3291	96.91%	5.39 (0.94)	1–6	5630	96.00%
**Perceived quality of implementers**												
1. The instructor(s) had a good mastery of the curriculum.	5.31 (0.91)	1–6	2236	96.60%	5.51 (0.81)	1–6	3319	97.70%	5.43 (0.86)	1–6	5633	97.27%
2. The instructor(s) was well prepared for the lessons.	5.37 (0.87)	1–6	2228	97.44%	5.54 (0.79)	1–6	3328	98.03%	5.48 (0.83)	1–6	5623	97.79%
3. The instructor(s)’ teaching skills were good.	5.38 (0.87)	1–6	2227	97.49%	5.53 (0.80)	1–6	3320	97.94%	5.47 (0.83)	1–6	5617	97.76%
4. The instructor(s) showed good professional attitudes.	5.40 (0.88)	1–6	2236	97.14%	5.55 (0.78)	1–6	3331	98.11%	5.49 (0.83)	1–6	5631	97.73%
5. The instructor(s) was very involved.	5.40 (0.87)	1–6	2232	97.67%	5.56 (0.78)	1–6	3334	98.29%	5.49 (0.82)	1–6	5624	98.04%
6. The instructor(s) encouraged students to participate in the activities.	5.42 (0.87)	1–6	2227	97.58%	5.57 (0.79)	1–6	3329	98.11%	5.51 (0.82)	1–6	5620	97.90%
7. The instructor(s) cared for the students.	5.38 (0.90)	1–6	2230	97.26%	5.52 (0.82)	1–6	3314	97.64%	5.47 (0.86)	1–6	5624	97.49%
8. The instructor(s) was ready to offer help to students when needed.	5.32 (0.92)	1–6	2233	96.87%	5.48 (0.86)	1–6	3300	97.37%	5.42 (0.89)	1–6	5622	97.17%
9. The instructor(s) had much interaction with the students.	5.36 (0.90)	1–6	2232	97.18%	5.51 (0.84)	1–6	3311	97.47%	5.45 (0.86)	1–6	5629	97.35%
10. Overall speaking, I have a very positive evaluation of the instructors.	5.42 (0.89)	1–6	2242	97.28%	5.57 (0.78)	1–6	3336	98.20%	5.51 (0.83)	1–6	5639	97.84%

All items were rated on a 6-point Likert-type scale, with 1 = strongly disagree, 2 = disagree, 3 = slightly disagree, 4 = slightly agree, 5 = agree, and 6 = strongly agree.

**Table 6:** Participants’ evaluations of the perceived program benefits.

Items	Junior Secondary (N = 2245)				Senior Secondary (N = 3409)				Overall (N = 5654)			
	Full Responses (Options 1–5)		Positive Responses (Options 3–5)		Full Responses (Options 1–5)		Positive Responses (Options 3–5)		Full Responses (Options 1–5)		Positive Responses (Options 3–5)	
	Mean (SD)	Range	n	%	Mean (SD)	Range	n	%	Mean (SD)	Range	n	%
1. It has strengthened my bonding with teachers, classmates and my family.	3.97 (1.05)	1–5	2235	90.02%	4.11 (1.03)	1–5	3130	91.98%	4.05 (1.04)	1–5	5638	91.20%
2. It has strengthened my resilience in adverse conditions.	4.02 (1.04)	1–5	2237	90.97%	4.13 (1.01)	1–5	3148	92.59%	4.09 (1.02)	1–5	5637	91.95%
3. It has enhanced my social competence.	4.09 (1.01)	1–5	2230	92.15%	4.16 (1.01)	1–5	3138	92.43%	4.13 (1.01)	1–5	5625	92.32%
4. It has improved my ability in handling and expressing my emotions.	4.09 (1.03)	1–5	2229	91.79%	4.18 (1.01)	1–5	3148	92.64%	4.15 (1.02)	1–5	5627	92.30%
5. It has enhanced my cognitive competence.	4.13 (0.98)	1–5	2230	93.23%	4.17 (0.99)	1–5	3174	93.55%	4.16 (0.99)	1–5	5623	93.42%
6. My ability to resist harmful influences has been improved.	4.21 (0.95)	1–5	2218	94.59%	4.22 (0.97)	1–5	3194	94.05%	4.21 (0.96)	1–5	5614	94.26%
7. It has strengthened my ability to distinguish between the good and the bad.	4.22 (0.94)	1–5	2229	95.02%	4.27 (0.94)	1–5	3229	95.17%	4.25 (0.94)	1–5	5622	95.11%
8. It has increased my competence in making sensible and wise choices.	4.18 (0.96)	1–5	2219	94.32%	4.24 (0.96)	1–5	3204	94.35%	4.22 (0.96)	1–5	5615	94.34%
9. It has helped me to have life reflections.	4.14 (1.04)	1–5	2227	91.96%	4.21 (1.00)	1–5	3161	93.08%	4.18 (1.02)	1–5	5623	92.64%
10. It has reinforced my self-confidence.	4.14 (1.05)	1–5	2228	91.47%	4.19 (1.03)	1–5	3124	92.07%	4.17 (1.04)	1–5	5621	91.83%
11. It has increased my self- awareness.	4.19 (0.99)	1–5	2222	93.38%	4.26 (0.95)	1–5	3219	94.82%	4.23 (0.97)	1–5	5617	94.25%
12. It has helped me to face the future with a positive attitude.	4.19 (1.00)	1–5	2225	93.08%	4.27 (0.96)	1–5	3215	94.61%	4.24 (0.98)	1–5	5623	94.01%
13. It has helped me to cultivate compassion and care about others.	4.15 (1.02)	1–5	2222	92.66%	4.21 (1.00)	1–5	3171	93.37%	4.18 (1.01)	1–5	5618	93.09%
14. It has encouraged me to care about the community.	3.97 (1.11)	1–5	2226	88.50%	4.03 (1.13)	1–5	3005	88.56%	4.01 (1.12)	1–5	5619	88.54%
15. It has promoted my sense of responsibility in serving the society.	4.17 (0.99)	1–5	2227	93.17%	4.24 (0.98)	1–5	3181	93.78%	4.21 (0.99)	1–5	5619	93.54%
16. It has enriched my overall development.	4.32 (0.93)	1–5	2238	95.13%	4.36 (0.90)	1–5	3268	96.06%	4.35 (0.91)	1–5	5640	95.69%

All items are rated on a 5-point Likert-type scale, with 1 = unhelpful, 2 = not very helpful, 3 = slightly helpful, 4 = helpful, and 5 = very helpful.

**Table 7:** Summary of the participants’ evaluations of satisfaction rating.

Items	Junior Secondary (N = 2245)				Senior Secondary (N = 3409)				Overall (N = 5654)			
	Full Responses		Positive Responses		Full Responses		Positive Responses		Full Responses		Positive Responses	
	Mean (SD)	Range	n	%	Mean (SD)	Range	n	%	Mean (SD)	Range	n	%
1. Will you suggest other students to participate in the program ^a^	3.45 (0.62)	1–4	2234	95.43%	3.49 (0.58)	1–4	3301	97.20%	3.47 (0.60)	1–4	5630	96.50%
2. Will you participate in similar programs in the future ^a^	3.45 (0.65)	1–4	2235	94.05%	3.52 (0.60)	1–4	3263	96.00%	3.49 (0.62)	1–4	5634	95.23%
3. Are you satisfied with the program ^b^	5.23 (0.98)	1–6	2227	97.67%	5.32 (0.91)	1–6	3322	98.49%	5.28 (0.94)	1–6	5600	98.16%

^a^ A 4-point Likert-type scale was used (1 = definitely will not, 2 = will not, 3 = will, 4 = definitely will). The positive responses are defined as a choice of Options 3–4. ^b^ A 6-point Likert-type scale was used (1 = very dissatisfied, 2 = moderately dissatisfied, 3 = slightly dissatisfied, 4 = slightly satisfied, 5 = moderately satisfied, 6 = very satisfied). The positive responses are defined as a choice of Options 4–6.

**Table 8:** Participants’ subjective outcome evaluations by grade level.

Factors	Junior		Senior		Comparison	
	M	SD	M	SD	F	*η_p_* ^2^
					6.05 ***	0.007
1. Perceived program quality	5.18	0.84	5.37	0.82	8.41 **	0.002
2. Perceived implementer quality	5.37	0.78	5.53	0.75	14.07 ***	0.003
3. Perceived program benefits	4.13	0.85	4.20	0.89	0.01	<0.001

** *p* < 0.01, *** *p* < 0.001. Covariates: age and gender.

## Data Availability

The raw data supporting the conclusions of this article will be made available by the authors upon request.
